# Supplementary material for: Fungal Jasmonate as a Novel Morphogenetic Signal for Pathogenesis
Source: J Fungi (Basel). 2021 Aug 26;7(9):693. doi: 10.3390/jof7090693 (PMC8471849; doi:10.3390/jof7090693)
Supplement: Supplementary file 1 [file jof-07-00693-s001.zip › jof-1357542-supplementary.pdf]

Liu et al., Supplementary Data

Figure S1

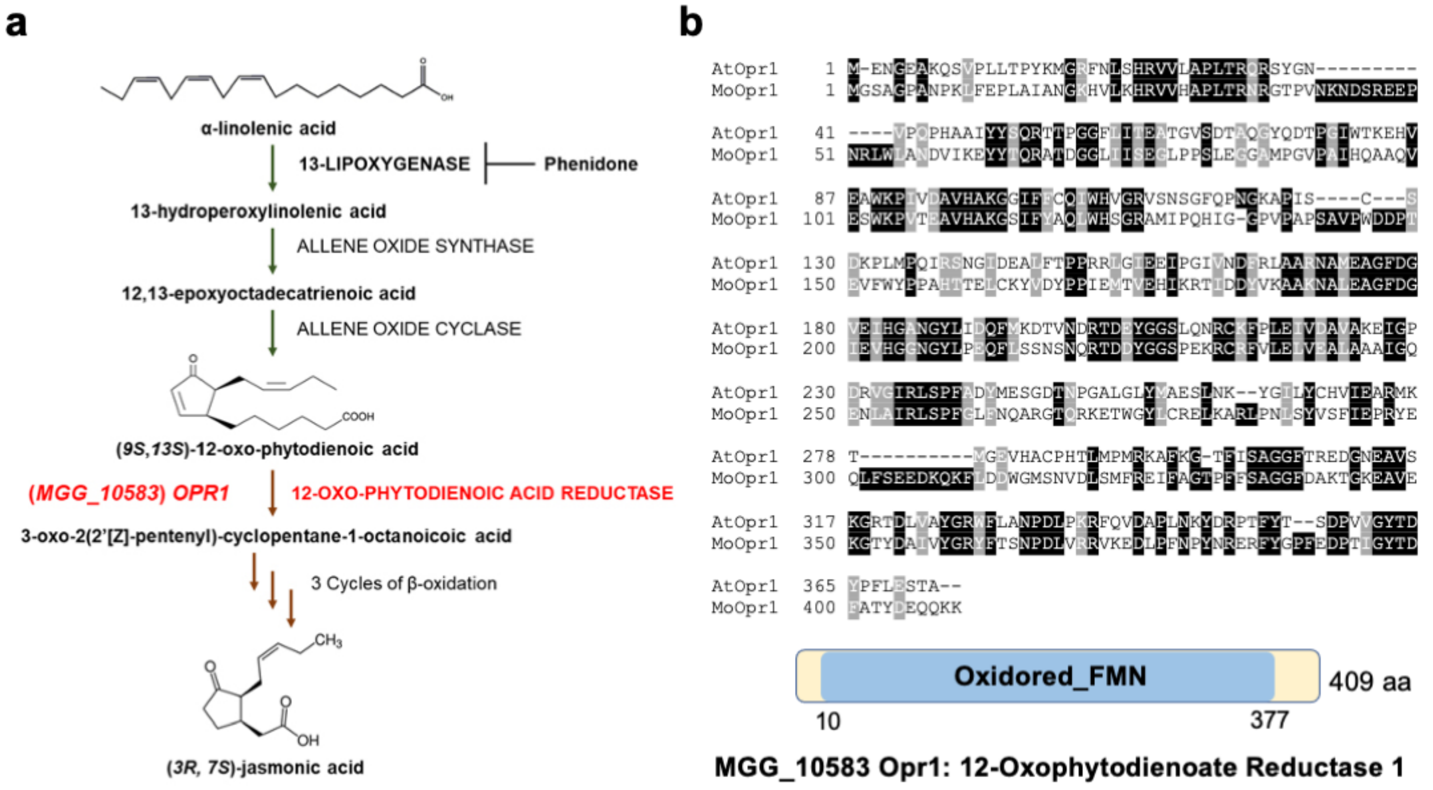

Figure S1 Prediction of JA biosynthesis pathway and identification of *OPR1* in *M. oryzae.*
(a) Schematic representation of the Jasmonic acid biosynthesis in plants with the predicted *OPR1* locus or function from the rice blast fungus *Magnaporthe oryzae* highlighted in red. First, polyunsaturated fatty acids, such as α-Linolenic acid, are synthesized from membrane components then secreted into plastids where 13-LOX, AOS, and AOC are functional. A pharmacological inhibitor, Phenidone, can block JA synthesis by inhibiting the lipoxygenase. Catalysed by the three specified enzymes, the essential intermediate substrate in JA biosynthesis, *cis*-OPDA, is then synthesized in the chloroplasts. The nascent *cis*-OPDA then migrates to peroxisomes, where it is reduced by OPDA reductase(s) and subsequently activated via a CoA esterification. The last step in such octadecatrienoic pathway is shortening of the carboxylic acid side chain via β-oxidation. Ultimately, *cis*-Jasmonic acid is biosynthesized in the peroxisome, and transported to the cytosol to be modified into different derivates. In *M oryzae*, we predicted the OPDA reductase *OPR1* (MGG_10583) based on AtOpr1 (AT1G76680), Uniprot annotation and Ensembl (https://fungi.ensembl.org/Magnaporthe_oryzae/Info/Index).
(b) Amino acid sequence comparison between *Magnaporthe* Opr1 and its ortholog in Arabidopsis. MoOpr1 protein (MGG_10583) was compared with AtOpr1 (AT1G76680), Residues that are conserved are highlighted in black. Clustal Omega and Boxshade were used for sequence alignment and graphical depiction, respectively. Detailed schematic illustration and domain organization of MoOpr1 MGG_10583 is presented in *M. oryzae*.

Figure S2

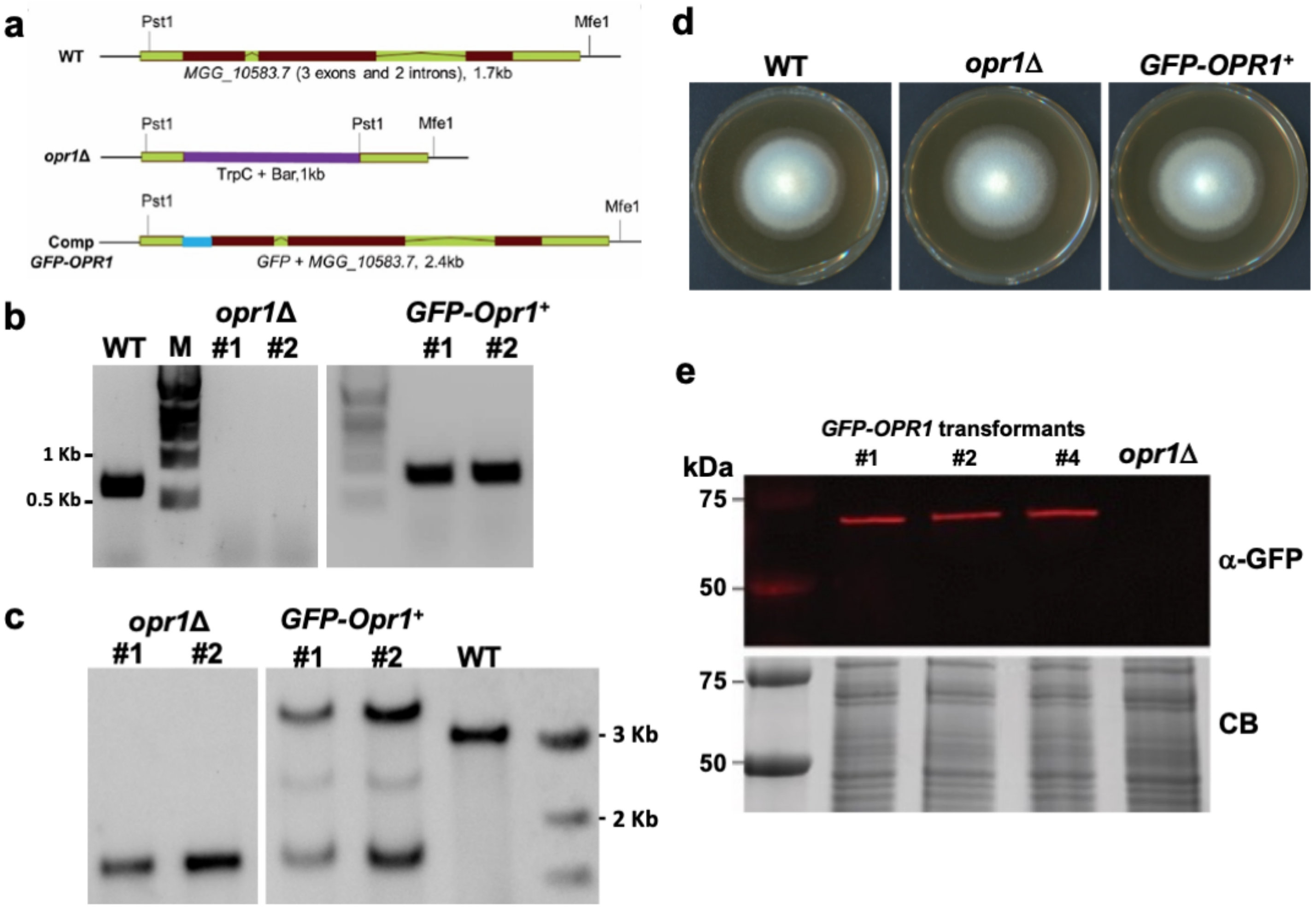

Figure S2 Generation of the gene deletion and complementation strains for *OPR1* in *M. oryzae.*
(a) Diagrammatic representation of the *OPR1* locus in blast fungus. Brown bars and green lines represent the exons and introns, respectively. Relevant restriction enzyme sites (Mfe1 and Pst1) are depicted, and Bar refers to the Bialaphos resistance marker cassette used to replace the *OPR1* coding region in the deletion strain. (b) Confirmation of *OPR* deletion mutant and the genetically complemented *opr1*Δ strain (*GFP-OPR1*$^+$) by locus-specific PCR. (c) Confirmation of *OPR* deletion mutant and the genetic complemented *opr1*Δ strain (*GFP-OPR1*$^+$) by Southern blot analysis. Genomic DNA from WT, *opr1*Δ (strain #1 and #2), and *opr1*Δ complemented strains (#1 and #2) were digested with PstI and MfeI and probed with the 1 kb *OPR1* 5'UTR fragment. The appearance of the 1.8 kb fragment in the deletion mutants and 3.1kb in wild-type indicated the specific *OPR1 ORF* replacement event. The complemented strain showed the 3.8 kb fragment while retaining the diagnostic 1.8 kb band. (d) Loss of *OPR1* does not affect vegetative growth or asexual development in the rice blast fungus. Comparative analysis of the growth characteristics, conidiation and colony morphology in the indicated strains of *M. oryzae* at 5 dpi (e) Western blot analysis confirming the presence of full-length GFP-Opr1 protein (72.8 kDa) in the 3 indicated transformants. The untagged *opr1*Δ strain served as the negative control. Total protein was extracted from 2-day old mycelial cultures, and 20 μg loaded per lane. The lower panel (CB) represents Coomassie brilliant blue staining as loading control.

Figure S3

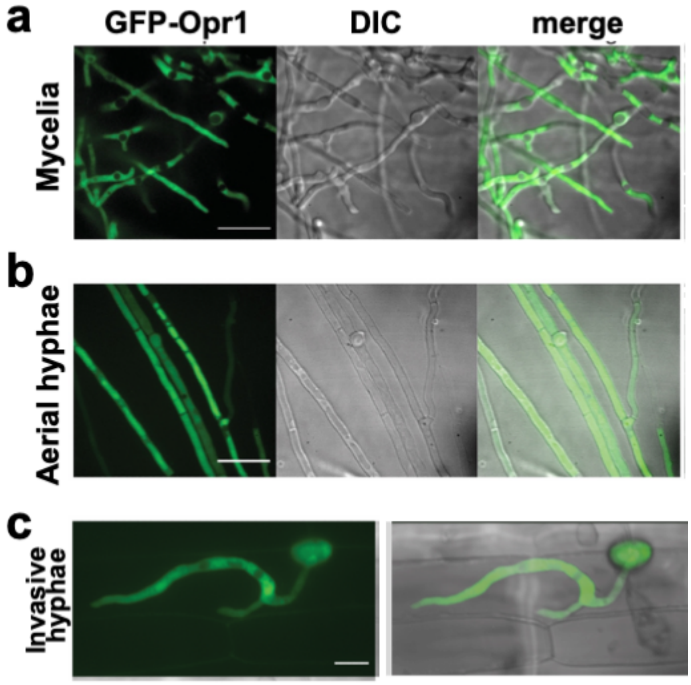

Figure S3 Subcellular localization of the GFP-Opr1 at different stages of vegetative, asexual, and pathogenic development in *M. oryzae.*
(a) and (b) GFP-Opr1 localizes to the cytosol during vegetative growth. Vegetative mycelia and aerial hyphae from the GFP-Opr1 strain were imaged at 3 dpi and 7 dpi, respectively. The size bar equals 10 µm. (c) Subcellular localization of GFP-Opr1 in conidia, developing germ tubes, and the incipient appressoria. Arrows indicate the punctate/vesicular localization of GFP-Opr1. Scale bar = 5 µm. (c) Subcellular localization of GFP-Opr1 during invasive *in planta* growth of *M. oryzae* in rice. The confocal images were captured at 26-28 hpi, which represents the early invasive growth phase in the host. Scale bar = 5 µm. Images shown are maximum intensity Z-projections of eight confocal stacks, each measuring 0.3 µm.

Figure S4

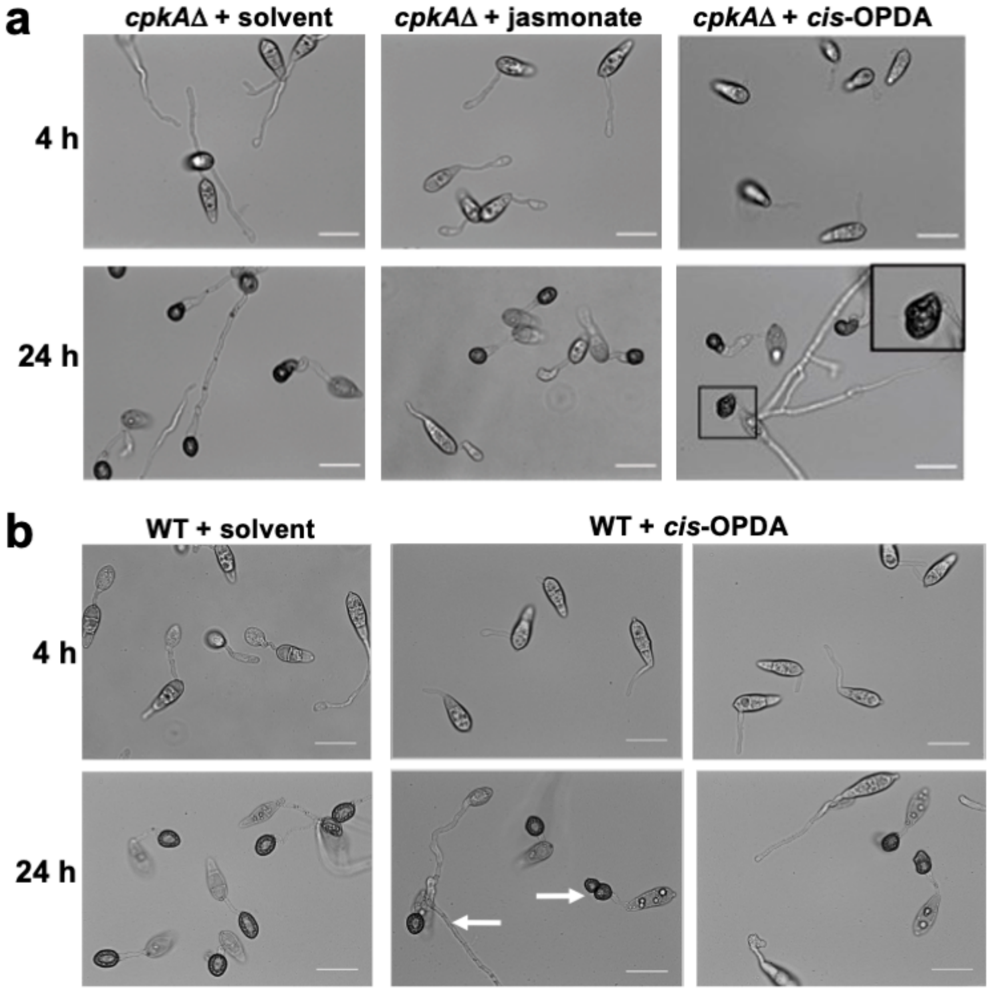

Figure S4 Exogenous cis-OPDA causes developmental defects in *cpkA*Δ and WT *M. oryzae*.
(a) *cis*-OPDA fails to suppress the germ tube and appressorial defects in the *cpkA*Δ mutant, and instead causes severe abnormalities in the infection structures therein and in WT strain too. Conidia from the *cpkA*Δ strain were treated with JA or *cis*-OPDA and germ tube development and appressorium initiation and formation analysed at 4 hpi and 24 hpi, respectively. Scale bar equals 10 micron. (b) Wild type conidia were treated with 10 µM *cis*-OPDA and pathogenic differentiation analysed at 4 hpi and 24 hpi, as above. Scale bars represent 10 micron. Arrow head indicates hooking stage / appressorium initiation, whereas arrows point to developmental defects in the OPDA-treated WT conidial germ tubes and appressoria. Please refer to the Bar graphs in Figure 8 for quantitative data analysis for the experiments described in (a) and (b) above. Although the oxylipin-treated germ tubes were shorter in length, the percentage of appressorium formation in such *cis*-OPDA treated *cpkA*Δ and WT conidia was significantly reduced. Data represent mean ± S.E from three biological replicates each using 300 conidia as sample size. *, $P<0.05$; **, $P<0.01$; ***, $P<0.001$ (unpaired two-tailed t-test; n=3 experiments).

Figure S5

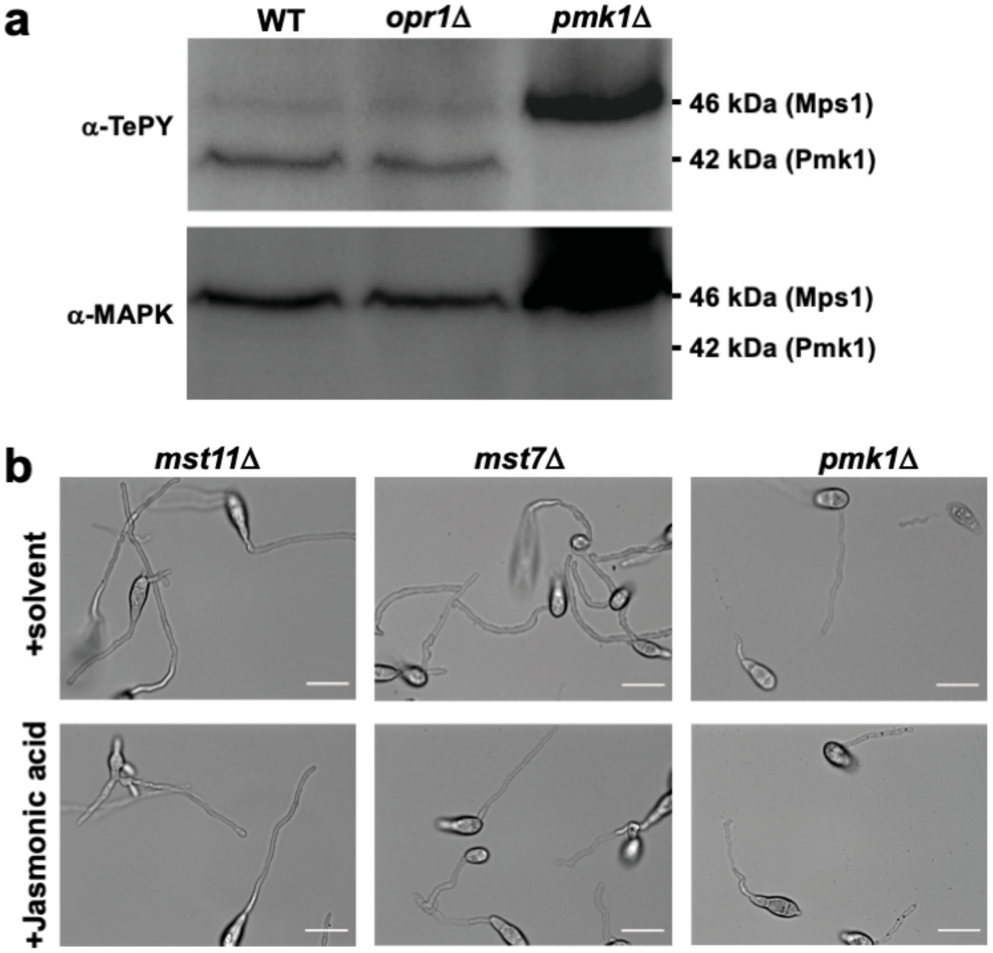

Figure S5 Loss of Opr1 does not affect Pmk1 MAPK signaling in *M. oryzae.*
(a) Western blot analyses showing the phosphorylation status of Pmk1 (42 kDa) and Mps1 (46 kDa) MAP kinases in the WT and *opr1*Δ mutant of *M. oryzae*. Total proteins were extracted from 2-day-old cultures of the indicated strains and analysed by western blotting with anti TePY or anti-MAPK antisera. The *pmk1*Δ strain served as a negative control. A representative image is shown for the experiment, which was repeated thrice.
(b) The oxylipin/Jasmonic acid signaling does not regulate the Pmk1-MAPK signaling in *M. oryzae.*
(c) Loss of Mst11 (MAPKKK), or Mst7 (MAPKK) or Pmk1 (MAPK) results in complete failure in appressorium formation, which cannot be reversed by exogenous JA (0.2 mM) added at 0 hpi. Conidia from the aforementioned strains were inoculated on the inductive surface in the presence (0.2 mM) or absence (solvent/mock) of jasmonic acid. The results were documented at 24 hpi. The experiment was repeated thrice with 300 conidia per sample in each instance. Scale bars represent 10 μm.

Figure S6

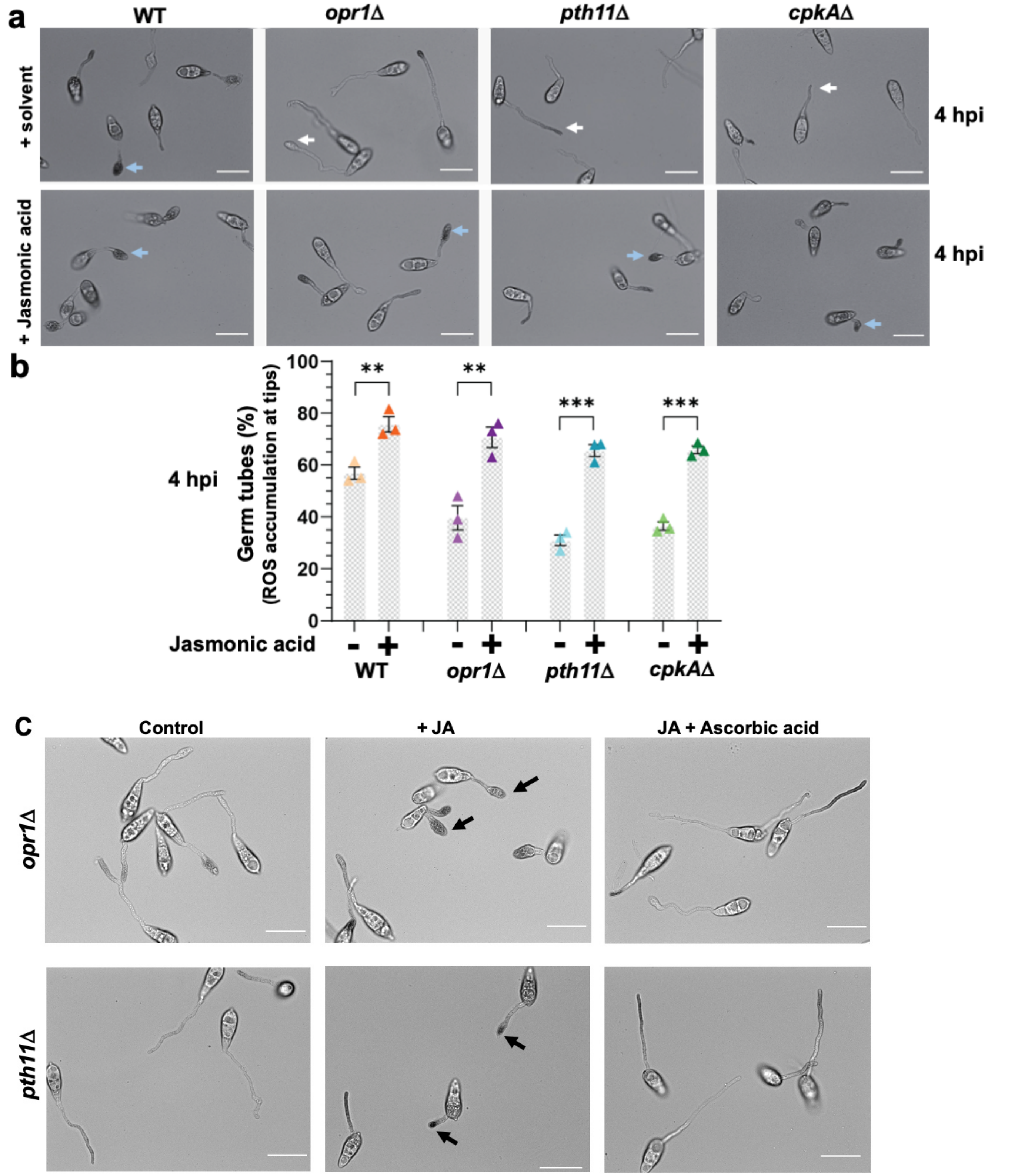

Figure S6 Fungal jasmonate is a key determinant of the cellular redox state during appressorium initiation in *M. oryzae.* (a) Loss of Opr1 or the key cAMP regulators (GPCR or CPKA) significantly reduces the overall ROS accumulation; and exogenous JA suppresses such redox defects in the aforementioned mutant strains of rice blast. Conidia from the indicated strains were germinated on inductive surface for 4 h in the presence or absence of 0.2 mM JA and stained with NBT to visualize the ROS at the tips of the germ tubes or hooking structures. (b) Bar charts depicting the detailed quantification of ROS accumulations at the germ tube tips of the indicated strains upon treatment with exogenous JA. Data represent mean±S.E from three biological replicates of the experiment each using 300 conidia per sample. (c) Antioxidant treatment negates the appressorium formation ability of JA in *opr1*Δ and *pth111*Δ. Conidia from the indicated mutant strains were treated with JA or JA + Ascorbic acid at 0 h and appressorium initiation (indicated by arrows) assessed at 4 hpi. Control refers to solvent/mock treatment. The samples were stained with NBT prior to microscopic analysis.

Supplementary Table S1 Oligonucleotide primers used in this study.

Restriction enzyme sites introduced for cloning purposes are depicted as underlined text.

Description	Identity	Primer sequence
***OPR1* deletion**	Opr1-5UTR-BamH1-F	TTTTAGGATCCTCTTGAATCGTGCCCCC
	Opr1-5UTR-Xba1-R	CCCCACTCTAGAGATTTTCTTTGGGGTAC
	Opr1-3UTR-Pst1-F	AACCAGTCGACCTGCAGGTACCATGCATAACC
	Opr1-3UTR-Pst1-R	GCTTGCATGCCTGCAGGCTTTATTTTATTCTGCCATC
**GFP-Opr1**	Opr1-Promoter-Kpn1-F	GGTGGTACCTTGCCTAACAAACAAAC
	Opr1-Promoter-Kpn1-R	GGTGGTACCGATTTTCTTTGGGGTAC
	GFP-Kpn1-F	TTGGGTACCATGGTGAGCAAGGGCGAGG
	GFP- (no stop codon)-BamH1-R	TTGGGATCCCTTGTACAGCTCGTCC
	Opr1-ORF-infusion-F	ACGAGCTGTACAAGGGATCCGGATCAGCCGGACCTGCC
	Opr1-3UTR-infusion-R	GACTCTAGAACTAGTGGATCCATCCCGCCGCTGCTGAACCGCACC
**Locus-specific PCR**	Opr1 ORF1.7kb-F	CGGTCCTTTTGAGGACCCTACG
